# Childhood body mass index and subsequent physician-diagnosed asthma: a systematic review and meta-analysis of prospective cohort studies

**DOI:** 10.1186/1471-2431-13-121

**Published:** 2013-08-13

**Authors:** Kathryn B Egan, Adrienne S Ettinger, Michael B Bracken

**Affiliations:** 1 Department of Chronic Disease Epidemiology, Yale School of Public Health, New Haven, CT, USA; 2 Center for Perinatal, Pediatric and Environmental Epidemiology, Yale University, 1 Church Street, 6th floor, New Haven, CT, 06510, USA; 3 Yale School of Medicine and Graduate School, New Haven, CT, USA

**Keywords:** Asthma, Overweight, Obesity, Body mass index, Body weight, Pediatric

## Abstract

**Background:**

Childhood asthma and obesity prevalence have increased in recent years suggesting a potential association. However, the direction of any association is poorly understood and the potential causal-relationship is unknown.

**Methods:**

We examined the association between overweight/obesity, defined by body mass index (BMI) <18 years of age, and subsequent physician-diagnosed incident asthma at least one year after BMI assessment. We sought to explore potential effect modification by sex. PubMed and Embase were searched using keywords and restricted to subjects aged 0–18 years. There were no date or language restrictions. From each study we extracted: authors, publication date, location, overweight/obesity definitions, asthma definitions, number of participants, recruitment duration, description of cohort, follow-up time, adjusted effect estimates (with 95% CI) and estimates of subgroup analysis.

**Results:**

Six prospective cohort studies which focused on children <18 years of age met criteria for inclusion. The combined risk ratio (RR) of overweight was associated with asthma (RR = 1.35; 95% CI = 1.15, 1.58). In boys, the combined RR of overweight on asthma was significant (RR = 1.41; 95% CI = 1.05, 1.88). For girls, when BMI was defined by Z-score, the combined RR of overweight on asthma was also significant (RR = 1.19; 95% CI = 1.06, 1.34). The combined risk ratio (RR) of obesity was associated with asthma in both boys and girls (RR = 1.50; 95% CI = 1.22, 1.83), in boys only (RR = 1.40; 95% CI = 1.01, 1.93) and in girls only (RR = 1.53; 95% CI = 1.09, 2.14).

**Conclusions:**

Overweight and, especially, obese children are at increased risk of subsequent physician diagnosed asthma in comparison to normal weight children. Except for sex, no studies reported any other potential effect modifiers. The observed sex effects were inconsistent.

## Background

Both asthma and obesity have increased in prevalence in recent years which suggests a possible association between the two conditions [1]. Childhood asthma/wheeze and obesity, measured by body mass index (BMI), have been linked in cross-sectional, case–control, and prospective epidemiologic studies. However, it is unknown whether the obesity and childhood physician-diagnosed asthma epidemics are causally related [2,3] and, if so, what is the direction of any potential association. Does obesity increase the risk for physician-diagnosed asthma, physician-diagnosed asthma increase the risk for obesity, or both? Obesity was implicated as a risk factor for asthma and wheeze in a meta-analysis of adult studies [4]. However, the sex-specific association between obesity, measured by age- and sex- specific BMI, and physician-diagnosed asthma in children less than 18 years of age is unclear.

Since 1980, rates of overweight (typically classified using International Obesity Task Force sex- and age- specific BMI standards [5]) have tripled among U.S. adolescents [6]. Epidemiological evidence suggests that the prevalence of asthma, a common childhood chronic illness [7,8], has also tripled since 1980 [9]. Asthma is characterized by airway inflammation, enhanced airway responsiveness to a variety of environmental stimuli, and reversible airway obstruction and is associated with significant medical and social morbidity [10,11]. Obesity in children, also a condition associated with significant medical and social morbidity, can lead to respiratory problems and has been linked to bronchial hyperactivity and reduced chest wall compliance [12]. Energy intake and energy expenditure, including appetite and metabolism are regulated by pro-inflammatory mediators that are derived from adipose tissue. These adipose-derived hormones include adiponectin [13], leptin [14,15], and, the cytokine-rich protein, resistin [16], among others. Adiponectin is exclusively secreted from adipose tissue and modulates both glucose regulation and fatty acid oxidation [13]. In adults, adiponectin is inversely correlated with body fat percentage but this relationship is not clear in children [17]. Leptin has a pro-inflammatory effect as it is responds specifically to adipose-derived inflammatory cytokines. These cytokines include resistin which affects insulin resistance, inflammation and energy homeostasis [16]. The pro-inflammatory role of leptin and resistin support the hypothesis that obesity may lead to new-onset (incident) asthma, which is also an inflammatory condition [14,15]. Some researchers hypothesize that asthma and obesity may have additive synergistic pro-inflammatory effects [18].

The prospective relationship between obesity, measured by body mass index (BMI), and asthma among children has not been evaluated in a systematic review. A meta-analysis of the prospective epidemiological data on the relationship between BMI and incident asthma among adults has been published [4]. The results suggest that the risk of adult incident-asthma increases with increasing BMI among both men and women although it is unclear how each study included in the meta-analysis defined incident asthma. Another meta-analysis of the effect of high birthweight (defined as ≥3.8 kg) or overweight during childhood (defined as BMI ≥85th percentile for age and sex, or Ponderal index ≥2.5 g/cm^3^ or ≥27 kg/m^3^) on future risk of asthma or wheeze reported that there is an increased risk of asthma and wheeze among those children with high birthweight (RR 1.2; 95% CI 1.1, 1.3) and among those with a high childhood body weight (RR 1.5; 95% CI 1.2, 1.8) [19]. A literature review focusing on the association between childhood nutritional status, defined by various measures of BMI, and risk of asthma or wheeze in adolescence concluded that childhood obesity may precede asthma and/or wheeze in adolescents [20]. This study reported no cumulative effect estimates of overweight/obesity on asthma. Since body weight alone, in contrast to BMI, does not take into account height and the presence of wheeze is not necessarily indicative of incident physician-diagnosed asthma, the focus of this updated meta-analysis is to assess the effect of overweight/obesity (defined by International Obesity Task Force sex- and age- specific BMI standards) during childhood on physician-diagnosed incident asthma at least one year after BMI measurement.

This systematic review focuses on the evidence for an association between childhood overweight/obesity and subsequent physician-diagnosed asthma. In addition, the review assesses the potential interaction between sex and BMI on asthma risk [21] since conflicting sex-specific associations have previously been reported [22,23]. We hypothesize that overweight/obese children are at increased risk of physician-diagnosed asthma and that this relationship differs by sex.

The objectives of this review were to:

1. Determine whether or not there is an association between overweight/obesity, as defined by BMI, before 18 years of age and subsequent physician-diagnosed incident asthma.

2. Assess whether or not there is effect modification on the risk of developing asthma among overweight/obese children by sex.

## Methods

### Study selection

The following inclusion and exclusion criteria were used to screen potentially eligible studies:

1) The primary objective was to investigate the relationship between overweight/obesity, as measured by BMI, and new-onset asthma in children and/or adolescents, at least 1 year after BMI measurement.

2) To establish temporality, the study used a prospective cohort design.

3) There were no language or date restrictions.

#### Types of participants

Identified study subjects under the age of 18 years, of both sexes and all ethnic groups, were included for review. Studies examining overweight/obesity as defined by child or adolescent BMI or BMI applied to pediatric growth charts were included for review. For BMI assessment, height and weight must have been measured by research staff, not collected from existing medical records, and then BMI calculated based on those measurements.

#### Types of exposure measures

BMI must have been categorized into age- and sex-specific Z-scores (continuous variable) and/or categorical overweight/obese variables. As the distribution of BMI at each age is skewed and variance increases with age, continuous age and sex-specific BMI Z-scores were used to represent each child’s sex-specific, age-adjusted level of adiposity [24]. Likewise, categorical overweight/obese variables based on age- and sex-specific percentiles on the CDC or International Obesity Task Force BMI growth charts can also be used [5,25].

Accordingly, overweight was defined as BMI at or above the 85th percentile and lower than the 95th percentile and obesity as BMI at or above the 95th percentile, for children of the same age and sex [26].

#### Types of outcome measures

Included studies must have used an asthma outcome definition that adhered to accepted diagnostic guidelines for childhood asthma [27]. Asthmatic subjects must have had new physician-diagnosed asthma at the time of study outcome assessment. Prescribed asthma medication usage was an acceptable outcome measure as it was assumed that physician-diagnosed asthma must precede use of prescription asthma medications. Over-the-counter asthma medication use was not assessed as information was unavailable in the included studies.

#### Primary outcomes

The primary outcome was the development of new physician-diagnosed asthma at least 1 year after height and weight were measured.

### Data sources

PubMed and Embase were searched in November 2012. There were no restrictions on publication date or language. Both were searched using the terms “overweight” and “asthma” or “obesity” and “asthma” or “body mass index” and “asthma” or “body weight” and “asthma.” The search was restricted to subjects aged 0–18 years. Reference lists of relevant studies identified in the electronic search were also checked to identify other potentially relevant studies.

### Data extraction and analysis

All identified studies were loaded into EndNote Web (2011). A single reviewer examined the titles for relevant articles and duplicates were identified and removed. Abstracts for remaining relevant studies were reviewed. Obviously ineligible studies (studies in adults or not involving asthma or obesity) were excluded. If it was not clear whether the study met the inclusion criteria, then the full text of the article was assessed. All studies excluded and reasons for exclusion were documented. Uncertainties were jointly discussed and resolved by consensus.

The following information, if available, was extracted from each included study: authors’ names, publication date, location of study, type of asthma, asthma medication use, and a brief description of the cohort, including: duration of recruitment, participant demographics, follow-up time, subgroup analyses (if any), crude and adjusted effect estimates reported and corresponding 95% confidence intervals, covariates included in analyses, and how overweight/obesity was assessed. The information was entered into Review Manager 5.1 [28]. Reviewers were not blinded to the names of authors, journals, or study institutions.

Studies were assessed for evidence of differential or non-differential selection bias using Cochrane Collaboration criteria [29]. Ascertainment bias was assessed by examining whether overweight or obese children were more likely to be screened and, subsequently, diagnosed with asthma. Attrition bias was determined by assessing completeness of follow-up, whether or not there were systematic differences in withdrawals, and/or differences in follow-up by exposure status.

Heterogeneity of studies in combined estimates was addressed by computing the I^2^ statistic which is designed to estimate the proportion of variation across studies due to differences rather than chance alone [30,31]. Low heterogeneity is defined as an I^2^ value 25-49%; moderate heterogeneity 50-74% and high heterogeneity ≥75%.

#### Data collection and synthesis

The crude relative risk (RR) or odds ratio (OR) for individual studies and 95% confidence intervals for all studies was computed and compared to reported crude RRs and ORs, when sufficient information was provided. If the computed effect estimates differed from the reported effect estimates, it was assumed that the reported effect estimate was an adjusted estimate. Comparisons were drawn between the crude and adjusted estimates. Estimates for the fixed effects model were calculated using the generic inverse variance method in which each study estimate is given a weight equal to the inverse of the variance of its effect estimate. Covariate-adjusted estimates were derived from all included studies.

Subgroup estimates were computed when at least two studies reported sufficient information to compute RRs for each subgroup. Data was available to examine the effects of age (young vs. older child) and sex (male vs. female). Publication bias could not be assessed using funnel plots because so few eligible prospective studies were eligible for inclusion [32].

## Results

**Figure 1:**
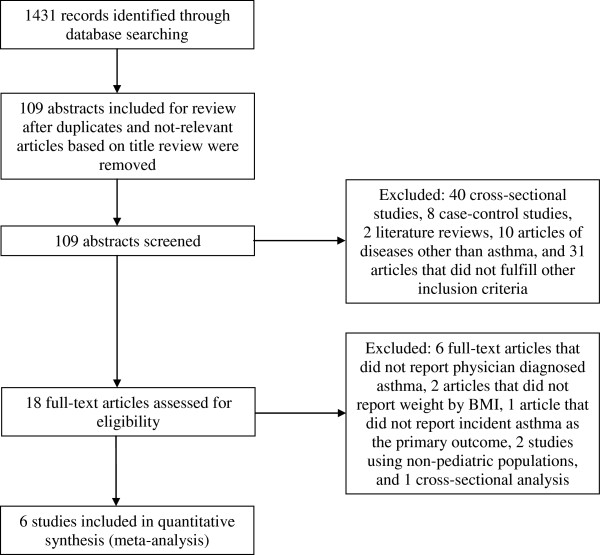
Study flow diagram: article search strategy results.

Figure 1 summarizes the number of articles identified and the search process. The initial literature search identified 1431 articles. Examination of the titles eliminated 1322 articles that did not meet the search criteria. Of 109 candidate abstracts reviewed, 91 studies were eliminated: 48 did not use a prospective cohort design, two were reviews, 10 investigated diseases other than asthma, and 31 did not fulfill other inclusion criteria. Eighteen studies were identified for full text review. Of those, twelve studies, listed in Table 1 by year, were excluded: six did not report physician-diagnosed asthma [33-38], two did not categorize overweight/obesity by BMI [39,40], one did not report incident asthma as the primary outcome [41], two reported only adult populations [42,43], and one used a cross-sectional analysis [44].

**Table 1:** Characteristics of excluded studies in full text review

**Study by year**	**Reason for exclusion**
*Castro-Rodriguez, 2001*[33]	Outcome measure defined as FEV and wheeze, rather than physician-diagnosed asthma.
*Chinn* &*Rona, 2001*[34]	Outcome measure not defined as physician-diagnosed asthma.
*Guerra et al., 2004*[39]	Exposure variable not defined as BMI.
*Hancox et al., 2004*[42]	Population over 18 years of age.
*Mai et al., 2005*[40]	Exposure variable not defined as BMI.
*Scholtens et al., 2009*[35]	Outcome measure defined as wheeze or dyspnea or medication use, rather than physician-diagnosed asthma.
*Taveras et al., 2008*[36]	Outcome measure defined as wheeze, rather than physician-diagnosed asthma. Exposure variable not defined as BMI.
*Tollefsen et al., 2007*[37]	Outcome measure defined as wheeze, rather than physician-diagnosed asthma.
*Wake et al., 2010*[41]	Incident asthma is not primary outcome of the study.
*Holguin et al., 2011*[43]	Population over 18 years of age.
*Suglia et al., 2011*[44]	Cross-sectional analysis.
*Magnusson et al., 2012*[38]	Outcome measure defined as at least 4 episodes of wheeze or 1 episode of wheeze with use of prescribed inhaled steroids, rather than physician-diagnosed asthma.

### Included studies

Six prospective cohort studies examining the association between BMI and subsequent physician-diagnosed asthma in children less than 18 years of age were included and are described in Tables 2 and 3. The results of these six studies are summarized in Table 4. Gilliland et al. found a significant association between overweight (BMI >85th percentile for sex and age) and incident asthma in both girls and boys combined (RR 1.52; 95% CI 1.14, 2.03) and in boys only (RR 2.06; 95% CI 1.33, 3.18) [45]. They found a significant association between obesity (BMI >95th percentile for sex and age) and incident asthma in both girls and boys combined (RR 1.60; 95% CI 1.08, 2.36) and in boys only (RR 2.29; 95% CI 1.35, 3.88) [45]. Gold et al. found significant associations between BMI Z-score and asthma (RR 1.43; 95% CI 1.09, 1.88) and between the highest BMI quintile and asthma (RR 2.24; 95% CI 1.14, 4.40) in girls only [46]. Mamun et al. found a borderline significant association in girls only between BMI Z-score at age 5 years and asthma at age 14 years (RR 1.14; 95% CI 1.00, 1.29). This was not a significant association in the total cohort or among boys only [47]. Mannino et al. found a significant association between overweight (BMI >85th percentile for sex and age) measured at 4–5 years of age and asthma in the total cohort (RR 1.80; 95% CI 1.20, 2.60) and in girls only (RR 2.30; 95% CI 1.20, 4.40) [48]. They found a significant association between overweight (BMI >85th percentile for sex and age) measured at baseline and asthma in boys only (RR 1.60; 95% CI 1.10, 2.40) [48]. Zhang et al. did not find any increased risk of incident asthma at age 6 or 8 years for normal weight (BMI < 85th percentile for sex and age) children at age 3 or 5 years [49]. Ho et al. found a significant association between obesity and incident asthma in girls only (RR 1.75; 95% CI 1.18, 2.61) [50].

**Table 2:** Cohort characteristics of included studies by year and author

**Study**	**N**	**Cohort**	**Follow-up period**	**Cohort description**
Gilliland et al., 2003 [45]	3792;	4th, 7th, and 10th grade public school, asthma-free students (aged 7–18 years)	Annually from 1993–1998 (4 years) or until high school graduation	288 developed asthma during follow-up; 58.1% Caucasian, 28.4% Hispanic, 4.8% Black, 5.6% Asian, 3.1% other; 24.2% overweight at baseline; 10.4% obese at baseline
	Girls: N = 1993			
	Boys: N = 1799			
Gold et al., 2003 [46]	9828;	U.S. Six-City Study: children aged 6–14 years	Annually from 1974–1979 (5–7 years)	90% were age 10 or younger at baseline; 3.4% of whites developed asthma with any wheeze during follow-up; 4.7% of blacks developed asthma with any wheeze during follow-up
	Girls: N = 4858			
	Boys: N = 4970			
Mamun et al., 2007 [47]	2812;	Australian birth cohort of 7223 mothers and children enrolled from 1981–1984; used 2812 child participants who had complete BMI and asthma at ages 5 and 14 years	Birth cohort assessed at first antenatal clinic visit between 1981–1984, 3–5 days post delivery, 6 months after birth, 5 and 14 years after birth	Mean BMI at age 5 years was 20.64; 8.13% had asthma at age 5 years; 22.28% had asthma in last 6 months at age 14 years
	Girls: N = 1340			
	Boys: N = 1472			
Mannino et al., 2006 [48]	4393;	U.S. Children born to women in the National Longitudinal Survey of Youth who first entered the cohort prior to age 2 years and were asthma-free at enrollment	14 years; 1986–2000; data collected every 2 years	218 developed asthma during follow-up; median age for asthma 7.6 years; 55.3% Caucasian, 25.6% Hispanic, 19.1% Black; 33.1% overweight at baseline
	Girls: N = 2218			
	Boys: N = 2175			
Zhang et al., 2010 [49]	259;	High-risk full-term newborns (birthweight > 2000 g) with at least 1 asthmatic/atopic parent	Nov 1998 - May 2000	56.4% male and 43.6% female; 86.8% Caucasian and 13.2% ethnic minority; 22% overweight at age 3 years
	Girls: N = 112 at 6 yrs;			
	Boys: N = 147 at 6 yrs;			
	238;			
	Girls N = 103 at 8 yrs; Boys N = 135 at 8 yrs			
Ho et al., 2011 [50]	4052;	Taiwanese adolescents with pre-asthmatic symptoms selected by non-physician staff from a cohort of 9546	12 month follow-up 1995-1996	10.9% boys and 14.1% girls developed asthma during follow-up; 13.0% overweight at baseline; 12.0% obese at baseline
	Girls: N = 1846			
	Boys: N = 2206			

**Table 3:** Exposure and outcome assessment of included studies by year and author

**Study**	**Obesity (exposure measure)**	**Asthma (outcome measure)**	**Confounder assessment**	**Subgroup analysis**	**Statistical methods**
Gilliland et al., 2003 [45]	Not overweight: ≤85th% vs. Overweight: >85th%;	New-onset physician diagnosed Asthma (child-report)	Age, sex, race, health insurance, community, parental history of asthma/allergies, birth weight, humidifier use, wheeze, allergy, team sports participation, smoking, household ETS, household pets and pests, puberty, and lung function	Sex	Cox proportional hazards
	Not obese: ≤95th% vs. obese: >95th%				
Gold et al., 2003 [46]	BMI by Z-score	New-onset doctor diagnosed Asthma with wheeze (parental-report)	Maternal smoking, air conditioner use, city of residence, child’s exact age, parental education level, single-parent household, only child status, and race	Sex, race, maternal smoking, age	Cox proportional hazards from BMI Z-score modeled as a time-dependent variable
	BMI by Quintile				annual updated BMI Z-scores included as a time-dependent variable
Mamun et al., 2007 [47]	BMI by Z-score at age 5 and 14 years	Asthma at ages 5 years (maternal-report) and age 14 years (self-report)		Sex	One-way ANOVA and F-test used for association between BMI and asthma; logistic regression
Mannino et al., 2006 [48]	Underweight: <25th%	New-onset asthma that limited child’s activity or required the use of medication or frequent attention from a doctor (parental-report)	Race/ethnicity, sex, poverty status, birthweight, and prenatal maternal smoking	Sex	Cox proportional hazards models
	Normal weight: 25th-84th%				
	Overweight/obese: ≥85th%				
Zhang et al., 2010 [49]	Age 3 years:	Incident asthma at age 6 years	Breast-feeding, sex, self-reported maternal asthma, dog and cat in household at birth, smoke exposure, day care attendance, having older children in household, and wheezing with rhinovirus infection.	Asthma at 6 years old, asthma at 8 years old	Logistic regression
	Low weight: <15th%				
	Average weight: 15th-84th%				
	High weight: >85th%				
	Age 5 years:	Incident asthma at age 8 years			
	Low weight: <15th%				
	Average weight: 15th-84th%				
	High weight: >85th%				
Ho et al., 2011 [50]	Underweight	New-onset physician diagnosed Asthma (self-report and parental-report)	Exercise, parental asthma, parental education, breastfeeding, air-conditioning usage, cigarette smoking, ETS, pet(s), and fungus/mold in the home	Sex	Mantel-Haenszel chi-square; Multivariable logistic regression
	Normal weight				
	Overweight				
	Obese				

**Table 4:** Effect estimates of overweight/obesity on incident physician-diagnosed asthma by sex

**Study**	**N**	**Years follow-up**	**Exposure**	**Outcome**	**Reference**	**Effect estimate**	**Total adjusted**	**Boys adjusted**	**Girls adjusted**
							**Effect estimate (95% CI)**	**Effect estimate (95% CI)**	**Effect estimate (95% CI)**
Gilliland et al., 2003^1^[45]	3792	4	Overweight (>85th%)	New-onset asthma	BMI ≤85th%	RR	1.52 (1.14, 2.03)	2.06 (1.33, 3.18)	1.25 (0.83, 1.88)
			Obesity (>95th%)	New-onset asthma	BMI ≤95th%		1.60 (1.08, 2.36)	2.29 (1.35, 3.88)	1.10 (0.60, 2.05)
Gold et al., 2003^2^[46]	9828	avg. 5	BMI at entry (Z-score)	Asthma with any wheeze	N/A	RR	Not reported	1.05 (0.83, 1.33)	1.43 (1.09, 1.88)
			BMI at entry Quintile 1	Asthma with any wheeze	Quintile 1			Reference	Reference
								0.42 (0.21, 0.83)	1.37 (0.69, 2.70)
			Quintile 2					1.01 (0.59, 1.73)	1.83 (0.95, 3.53)
			Quintile 3					1.13 (0.67, 1.91)	1.76 (0.89, 3.46)
			Quintile 4					1.04 (0.60, 1.82)	2.24 (1.14, 4.40)
Mamun et al., 2007^3^[47]	2812	9	Z-score of BMI at age 5 years	Asthma at age 14	N/A	OR	1.01 (0.92, 1.11)	0.87 (0.75, 1.01)	1.14 (1.00, 1.29)
Mannino et al., 2006^4^[48]	4393	avg. 6	Overweight/Obese (≥85th%) at baseline	Incident asthma	BMI 25th-84th%	HR	1.2 (0.9, 1.7)	1.6 (1.1, 2.4)	0.8 (0.5, 1.4)
			Overweight/Obese (≥85th%) 4-5 years old				1.8 (1.2, 2.6)	1.4 (0.9, 2.6)	2.3 (1.2, 4.4)
			Overweight/Obese (≥85th%) 6-7 years old				1.3 (0.7, 2.1)	1.6 (0.8, 3.2)	0.9 (0.4, 2.0)
			Overweight/Obese (≥85th%) 8-9 years old				1.2 (0.6, 2.4)	1.2 (0.5, 3.2)	1.3 (0.5, 3.4)
			always Overweight/obese				2.0 (1.3, 3.1)	2.4 (1.4, 4.4)	1.5 (0.7, 2.9)
Zhang et al., 2010^5^[49]	259	3	Normal Weight (<85th%) at 3 years	Incident asthma at 6 years	Overweight (≥85th%)	OR	1.04 (0.50, 2.15)	Not reported	
		5		Incident asthma at 8 years			1.51 (0.69, 3.30)		
	238	1	Normal Weight (<85th%) at 5 years	Incident asthma at 6 years	Overweight (≥85th%)		0.91 (0.45, 1.88)		
		3		Incident asthma at 8 years			1.31 (0.61, 2.82)		
Ho et al., 2011^6^[50]	4052	1	Underweight	Physician-diagnosed asthma	Normal weight	OR	Not reported	0.76 (0.49, 1.18)	0.79 (0.49, 1.26)
			Overweight					1.02 (0.67, 1.55)	1.12 (0.76, 1.67)
			Obese					1.04 (0.69, 1.56)	1.75 (1.18, 2.61)
			Change in BMI (per unit of BMI increase in kg/m^2^)		N/A			1.02 (0.91, 1.14)	1.01 (0.90, 1.14)

^1^Gilliland et al., 2003: RR adjusted for race/ethnicity, community, sex-specific effects of allergy, and history of wheezing.

^2^Gold et al., 2003: RR adjusted for city, race, age, parental education, air-conditioning, being an only child, single-parent family, and maternal smoking at entry.

^3^Mamun et al., 2007: OR adjusted for maternal age at birth, education, income, maternal smoking, birth weight, and breastfeeding.

^4^Mannino et al., 2006: HR adjusted for sex and prenatal maternal smoking.

^5^Zhang et al., 2010: OR adjusted for sex, maternal asthma, environmental factors during the first year, and wheezing with rhinovirus infection.

^6^Ho et al., 2011: OR adjusted for exercise frequency, parents with asthma, parental education, breastfeeding, pet(s), household fungus/mold, air-conditioning usage, smoking and environmental tobacco smoke exposure.

### Combined effect estimates

#### Overweight

**Figure 2:**
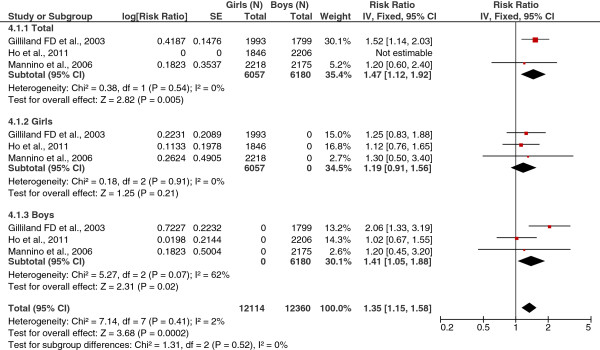
Forest plot of overweight/obesity (categorical variable) and physician-diagnosed asthma (total and by sex).

As shown in Figure 2, the combined RR for the three studies examining overweight (BMI ≥85th percentile for age and sex) and physician-diagnosed asthma in boys and girls combined was significant (RR = 1.35; 95% CI = 1.15, 1.58; I^2^ = 2%). When the analyses were stratified by sex, the combined RR for overweight and physician-diagnosed asthma in boys was significant (RR = 1.41; 95% CI = 1.05, 1.88; I^2^ = 62%). No significant association was observed for girls.

#### Obesity

**Figure 3:**
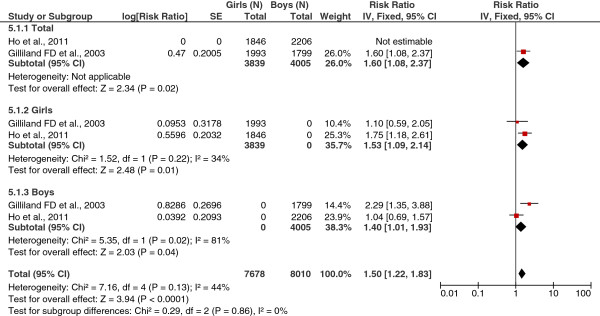
Forest plot of obesity (categorical variable) and physician-diagnosed asthma (total and by sex).

As shown in Figure 3, the combined RR of obese (BMI ≥95th percentile for age and sex) and physician-diagnosed asthma in children was significant (RR = 1.50; 95% CI = 1.22, 1.83; I^2^ = 44%). When the analyses were stratified by sex, obesity remained significant in both boys (RR = 1.40; 95% CI = 1.01, 1.93; I^2^ = 81%) and girls (RR = 1.53; 95% CI = 1.09, 2.14; I^2^ = 34%).

#### BMI Z-scores

**Figure 4:**
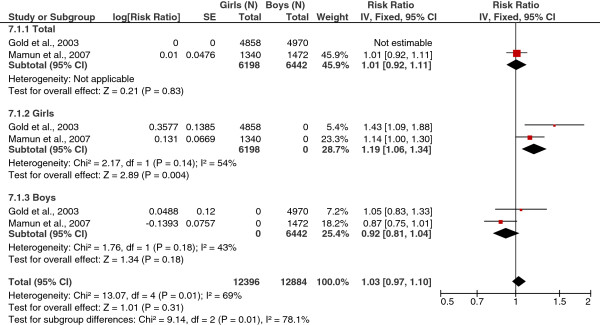
Forest plot of BMI z-score (continuous variable) vs. physician-diagnosed asthma (total and by sex).

As shown in Figure 4, the association between BMI, measured by continuous Z-scores, and physician-diagnosed asthma was significant in girls (RR = 1.19; 95% CI = 1.06, 1.34; I^2^ = 54%). BMI Z-score was not significantly associated with asthma in the combined cohort or among boys alone. For the fixed effects models, all combined RRs are listed in Table 5.

**Table 5:** Fixed effects model data and analyses

**Exposure variable**	**Outcome or subgroup**	**Studies**	**Participants**	**Statistical method**	**Effect estimate**
Overweight/Obesity	1.1 Overweight vs. physician-diagnosed asthma at age 9+ (Total)	3	12237	Risk ratio (IV, Fixed, 95% CI)	1.35 [1.15, 1.58]
	1.2 Obese vs. physician-diagnosed asthma in children ages 8–18 years (Total)	2	15688	Risk ratio (IV, Fixed, 95% CI)	1.50 [1.22, 1.83]
	1.3 Overweight vs. physician-diagnosed asthma at age 9+ (Boys)	3	6180	Risk ratio (IV, Fixed, 95% CI)	1.41 [1.05, 1.88]
	1.4 Overweight vs. physician-diagnosed asthma at age 9+ (Girls)	3	6057	Risk ratio (IV, Fixed, 95% CI)	1.19 [0.91, 1.56]
	1.5 Obese vs. physician-diagnosed asthma in children ages 8–18 years (Boys)	2	4005	Risk ratio (IV, Fixed, 95% CI)	1.40 [1.01, 1.93]
	1.6 Obese vs. physician-diagnosed asthma in children ages 8–18 years (Girls)	2	3839	Risk ratio (IV, Fixed, 95% CI)	1.53 [1.09, 2.14]
	1.7 BMI at age 3 vs. incident asthma at 6 years of age	1	249	Odds ratio (IV, Fixed, 95% CI)	1.04 [0.50, 2.16]
	1.8 BMI at age 3 vs. incident asthma at 8 years of age	1	229	Odds ratio (IV, Fixed, 95% CI)	1.51 [0.69, 3.30]
	1.9 BMI at age 5 vs. incident asthma at 6 years of age	1	255	Odds ratio (IV, Fixed, 95% CI)	0.91 [0.45, 1.84]
	1.10 BMI at age 5 vs. incident asthma at 8 years of age	1	235	Odds ratio (IV, Fixed, 95% CI)	1.31 [0.61, 2.81]
BMI Z-score	2.1 BMI Z-score vs. any physician diagnosed asthma (Total)	2	25280	Risk ratio (IV, Fixed, 95% CI)	1.03 (0.97, 1.10)
	2.2 BMI Z-score vs. any physician diagnosed asthma (Boys)	2	6442	Risk ratio (IV, Fixed, 95% CI)	0.92 [0.81, 1.04]
	2.3 BMI Z-score vs. any physician diagnosed asthma (Girls)	2	6198	Risk ratio (IV, Fixed, 95% CI)	1.19 [1.06, 1.34]
	2.4 Asthma at age 14 years (Total)	1	2812	Odds ratio (IV, Fixed, 95% CI)	1.01 [0.92, 1.11]
	2.5 Asthma at age 14 years (Boys)	1	1472	Odds ratio (IV, Fixed, 95% CI)	0.87 [0.75, 1.01]
	2.6 Asthma at age 14 years (Girls)	1	1340	Odds ratio (IV, Fixed, 95% CI)	1.14 [1.00, 1.30]
BMI Z-score	3.1 BMI at entry vs. asthma with any wheeze (Boys)	1	4970	Risk ratio (IV, Fixed, 95% CI)	1.05 [0.83, 1.33]
	3.2 BMI at entry vs. asthma with any wheeze (Girls)	1	4858	Risk ratio (IV, Fixed, 95% CI)	1.43 [1.09, 1.88]

Data were not available to measure the effects of any other variables as potential only available effect modifiers of the obesity/overweight and asthma associations.

### Potential sources of bias

Table 6 lists the risk of bias in each study.

**Table 6:** Risk of bias table

**Bias**	**Studies**	**Authors’ judgment**	**Support for judgment**
Incomplete outcome data (attrition bias)	*Gilliland et al., 2003*[45]*; Gold et al., 2003*[46]*; Mannino et al., 2006*[48]	Unclear risk	Number of participants lost to follow-up not reported
	*Mamun et al., 2007*[47]	High risk	46.5% of original cohort lost to follow-up by age 5 years; 59.6% of original cohort has incomplete data at either age 5 years or 14 years and was excluded from analysis
	*Zhang et al., 2010*[49]	Low risk	89.6% of original cohort sampled at 6 year visit; 82.4% of original cohort sampled at 8 year visit
	*Ho et al., 2011*[50]	Unclear risk	82% of selected original cohort with complete data at 12 month follow-up
Ascertainment bias	*Gilliland et al., 2003*[45]*; Gold et al., 2003*[46]*; Mamun et al., 2007*[47]*; Mannino et al., 2006*[48]*; Zhang et al., 2010*[49]*; Ho et al., 2011*[50]	Unclear risk	Unclear if children who were overweight or obese were more likely to be diagnosed with asthma
Differential misclassification of exposure bias	*Mannino et al., 2006*[48]	High risk	Only 69% of height and 61% of the weights were measured

#### Ascertainment bias

Given similar presenting symptoms, it is unclear whether or not overweight/obese children were more likely than normal weight children to be diagnosed with asthma in any of the included studies, so this bias could not be assessed.

#### Classification of exposure bias

Mannino et al. has a risk of differential misclassification of BMI as only 69% of height and 61% of weight measurements were by study personnel [48]. The remaining values were self-report height and weight which are known to be over- and under- estimated, respectively [51]. The other five studies do not appear to be at risk.

#### Attrition bias

Gilliland et al., Gold et al., and Mannino et al. did not report the number of participants lost to follow-up [45,46,48]. Therefore, it is unclear whether attrition bias is present in these studies. Mamun et al. had a high risk of attrition bias as 46.5% of the original cohort was lost to follow-up by age 5 years and 59.6% of original cohort had incomplete data at either age 5 or 14 years and was excluded from the analysis [47]. Zhang et al. has low risk of attrition bias as the authors sampled 89.6% of the original cohort at the 6 year visit and 82.4% of the original cohort at the 8 year visit [49]. Ho et al. also has low risk of attrition bias as 82% of the selected original cohort had complete data at 12 month follow-up [50].

## Discussion

Overweight and/or obese children were at increased risk of physician-diagnosed asthma (RR = 1.35; 95% CI = 1.15, 1.58). When stratified by sex, overweight boys remained at significantly increased risk of asthma. The overall increased risk appears to be more pronounced in the children who were obese (RR = 1.50; 95% CI = 1.22, 1.83). When stratified by sex, this association remained significant in both boys, although with high heterogeneity that could not be explained, and girls. When BMI was defined using Z-scores, only girls were at significantly increased risk of physician-diagnosed asthma (RR = 1.19; 95% CI = 1.06, 1.34). The effect of obesity (a fifty percent increased risk of asthma) was observed in both boys and girls suggesting that sex does not appear to be a modifier of the effect of childhood obesity and asthma. However, overweight boys were at increased risk of asthma in comparison to girls and when BMI was measured by z-score girls were at increased risk of asthma in comparison to boys. This suggests that the type of overweight/obesity BMI measurement could influence study results. BMI alone cannot distinguish between fat and muscle mass and may, therefore, inadequately reflect fat distribution which can differ by sex. In children, this may under- or over-estimate obesity as wide variations in body fat distribution can occur within the same BMI percentile group [52]. BMI assessment could also potentially be modified by timing of puberty. Sex hormonal fluctuations, a biological phenomenon occurring during puberty known to influence asthma, especially among girls during the menstrual cycle, may explain the relationship between asthma and puberty [53]. Additionally, leptin has been proposed to be peripherally involved in respiratory function regulation and sexual maturation [54]. Included studies were either of pre-pubescent children or did not control for puberty or body fat distribution and, therefore, further research is needed to tease apart this complex association and determine whether these factors could confound the sex-specific overweight/obesity and asthma association.

The evidence for these associations, assessed in this systematic review using the most valid methodology available, supports the hypothesis that overweight, and especially obese, children are at significantly increased risk of incident physician-diagnosed asthma. However, additional high quality research is needed to confirm this finding as there are a limited number of prospective studies focusing on this issue. In two studies [45,49], where BMI was analyzed as a categorical variable, the combining of normal weight and underweight children into one reference category may have led to an underestimation of the true effect. In two other studies [46,47], where BMI was assessed as a continuous Z-score, the association between BMI and asthma was only statistically significant in girls and had a moderate level of heterogeneity (54%). Other possible subgroup analyses of potential effect modifiers, such as: race/ethnicity, geographic location, family history of asthma and/or allergy, *in utero* exposure to maternal smoking, and birthweight, were not possible due to insufficient reporting of information, a limited number of eligible studies, and variations in duration of follow-up and child ages. Whether or not the observed association between obesity and asthma is causal, or it results from a risk factor common to both conditions cannot be ascertained from this analysis.

### Quality of the evidence

Assessing the quality of evidence was difficult due to a number of limiting factors. First, there was inadequate reporting of unadjusted effect estimates. None of the included articles listed the unadjusted effect estimates and few included adequate data to calculate them. Second, recruitment methods and characteristics of the study populations were not always complete. Detailed inclusion and exclusion criteria for each cohort were rarely provided. Third, study participation rates and losses to follow-up were only included in three articles [47,49,50]. Due to the inadequacy of reporting, it was difficult to assess: how and when participants in each cohort were recruited, exact ages at which exposure and outcome were assessed, and the overall internal validity and generalizability of each study. None of the studies reported on diet or physical activity levels.

### Agreements and disagreements with other studies or reviews

Results of this meta-analysis: that overweight and, particularly, obese children are at a 40-50% increased risk of physician-diagnosed asthma, support other published reviews examining the association between overweight/obesity and asthma and/or wheeze. Previous meta-analyses of adults [4] and children [19] have been published. Flaherman and Rutherford [19] analyzed wheeze as a primary outcome in addition to asthma; however, that assessment differs from this meta-analysis as we only included studies that reported physician-diagnosed asthma as the primary outcome measure. Wheeze is an unreliable indicator of physician-diagnosed asthma [55]. Further, Flaherman and Rutherford [19] defined overweight/obesity as high birthweight and/or body weight instead of using age- and sex-specific BMI.

Obese girls and boys were at significantly increased risk of subsequent physician-diagnosed asthma suggesting that sex is not a modifier of the effect of obesity, defined as age- and sex-specific BMI ≥95th%, on subsequent asthma diagnosis. However, when overweight/obesity was assessed via a continuous BMI Z-score, girls, but not boys, were at significantly increased risk of physician-diagnosed asthma. The opposite was true when overweight was categorically defined as age- and sex-specific BMI ≥85th%. In this case, boys were at significantly increased risk of asthma. This suggests that for the sex-specific overweight-to-asthma association the classification used to define the exposure is important and may influence conclusions drawn. It is also unclear whether the sex-specific associations observed could be influenced by timing of puberty, which we were unable to assess. Interestingly, Beuther and Sutherland reported a significant association between adult overweight/obesity, defined as BMI ≥ 25 kg/m^2^, and asthma incidence for both men (OR = 1.46, 95% CI 1.05, 2.02) and women (OR = 1.68; 95% CI = 1.45, 1.94) [4]. This further supports our findings which suggest that the overweight/obese variable classification may be influential in assessing the sex-specific overweight/obese and asthma association as different classifications may yield varying results.

## Conclusion

This systematic review provides an up-to-date assessment of the published prospective studies among children on the association between overweight/obesity and subsequent incident physician-diagnosed asthma in childhood. In children, the association between overweight and physician-diagnosed asthma appears to vary by sex based on how BMI is classified but these effects are inconsistent and limited to a few studies. Future research needs to better account for different durations of follow-up, ages at baseline and assessment, and assessment of obesity (categorical or continuous variables). In addition, further research should investigate the potential impact of other risk factors, such as antibiotic exposure that may be independently associated with risk of asthma and obesity. It is important to understand whether asthma and obesity are causally associated and, if so, the directionality of the causal pathway or whether both are outcomes from a common exposure.

## Competing interests

The authors declare that they have no competing interests.

## Authors’ contributions

KBE and MBB were responsible for planning the meta-analysis. KBE and ASE carried out final literature searches with MBB’s assistance. Data entry was carried out by KBE and checked by MBB. Interpretation of published data and the appropriate methods for derivation of RRs were discussed by all authors. The statistical analysis was conducted by KBE and discussed with MBB. KBE drafted the paper, which was then critically reviewed by MBB and ASE. All authors read and approved the final manuscript.

## Pre-publication history

The pre-publication history for this paper can be accessed here:

http://www.biomedcentral.com/1471-2431/13/121/prepub
